# A scoping review of the impacts of forest cover dynamics on acari-borne diseases: Beyond forest fragmentation

**DOI:** 10.1016/j.heliyon.2025.e41893

**Published:** 2025-01-11

**Authors:** Nolwenn Blache, Karine Chalvet-Monfray, Christophe Déprés, Serge Morand

**Affiliations:** a Université Clermont Auvergne, UMR Territoires, VetAgro Sup, 89 Av. de l'Europe, 63370 Lempdes, France; b Université de Lyon, UMR EPIA, INRAE, VetAgro Sup, 69280 Marcy l’Etoile, France; c IRL HealthDEEP CNRS – Kasetsart University – Mahidol University, Faculty of Veterinary Technology, Kasetsart University, Bangkok, Thailand; d Department of Social and Environmental Medicine, Faculty of Tropical Medicine, Mahidol University, Bangkok, Thailand

**Keywords:** Tick, Chigger mite, Acari, Vector-borne disease, Forest, Forest dynamics, Fragmentation, Conversion, Reforestation, Deforestation

## Abstract

Forest cover has undergone significant changes accelerating over recent decades. Acari vectors such as ticks and chigger mites are intricately linked to forest ecosystems because of the suitable hosts and microclimates provided. However, the implications of forest cover change on Acari vectors and their pathogens remain poorly understood. This study investigates the impacts of forest cover dynamics on Acari-borne diseases risk worldwide through a comprehensive review of the literature.

We conducted a scoping review following the PRISMA Method to retrieve citations related to forest cover dynamics and Acari-borne diseases. Eligibility criteria were predefined and relevant data were extracted from selected articles. The analysis employed a descriptive approach and thematic narrative synthesis.

Our review revealed that the influence of forest cover dynamics on Acari-borne diseases and vectors was predominantly discussed within a Western context, especially regarding *Ixodes* ticks and Lyme disease. Four types of forest cover dynamics have been identified in the literature: deforestation, fragmentation, conversion and reforestation. However, there was no consensus on their impacts on vectors and pathogens. Studies reported conflicting findings including: protective or risk effects, nonlinear relationships, dependent effects influenced by additional factors altering relationships or no significant effects. Although, there is limited empirical evidence on tropical contexts as well as for reforestation and conversion dynamics. Differences in results trends emerge according to the article type, with literature reviews often overestimating the dilution effect, which assumes that species diversity reduces disease risk, observed in empirical research. Finally, our review identifies a notable absence of studies on scrub typhus.

This scoping review provides a novel and comprehensive overview of global literature on the impacts of forest cover dynamics on Acari vectors and the infectious agents they transmit. It highlights the need for future research targeting specific forest cover dynamics on chigger mite vectors in a tropical context.

## Graphical abstract

**Figure:**
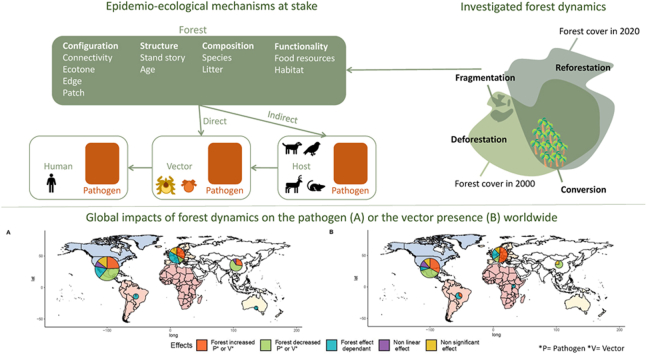

## 1 Introduction

The subclass Acari wich includes ticks and chigger mites plays a crucial role in the transmission of several vector-borne diseases [1,2]. The spread of tick-borne diseases, such as Lyme disease, anaplasmosis and babesiosis, is a growing concern with significant implications for both humans and domestic animals [3,4]. Concurrently, chigger mites are causing a neglected tropical disease known as scrub typhus. In endemic regions of Thailand, up to 77 % of rural human communities have been affected by this disease [5].

Ticks are recognized as some of the most common vectors of infectious diseases in temperate areas across North America, Europe, and Asia [6]. They are classified into two distinct families, the Ixodidae known as hard ticks and the Argasidae known as soft ticks. Most tick-borne diseases are caused by ticks belonging to the Ixodidae family, with various species having medical and veterinary importance [2]. Argasidae ticks stand out for their endophilic or nidicolous nature. These ticks colonize burrows or nests, making them more challenging to monitor. Given their nidicolous behaviour, soft ticks have ubiquitous habitats that depend more on hosts and their related microhabitats availability than on the microclimate [7]. This scoping review focuses only on hard ticks and chigger mites. Among the Ixodidae family, *Ixodes* is the primary genus responsible for disease transmission in temperate forests, but other genera can be implicated worldwide such as *Dermacentor*, *Rhipicephalus*, *Amblyomma*, *Haemaphysalis*, *Hyalomma*, and Rhipicentor. Due to wide variety of ticks, they can transmit numerous pathogens, including the most common: Borrelia burgdorferi sensu lato, the causative agent of Lyme disease, Anaplasma spp., Babesia spp., Rickettsia spp. etc. [2]. Chigger mites are globally distributed as well but show a higher diversity in tropical, subtropical and southern temperate zones [8]. They belong to the super-family Trombiculidae and species of the genus *Leptotrombidium* are the vectors of scrub typhus in Asia [9]. The causative agent of scrub typhus is *Orientia tsutsugamushi* belonging to the family Rickettsiaceae and formerly classified as *Rickettsia*. *Orientia tsutsugamushi* is endemic to the so-called Tsutsugamushi Triangle, which encompasses North Japan, Korea, Southeast Asia, Southwest Pacific, and East Russia. However, some human cases have also been reported in Chile and the vector in Africa [10]. *Orientia tsutsugamushi* is the only agent confirmed to be transmitted by chigger bites [8] whereas evidence of protist, viral and bacterial agents transmitted through tick bites has been referenced [11].

Ectoparasites like ticks and chiggers do not live in isolation, but cycle through stages of free living in the environment and stages of feeding on vertebrate hosts. They transmit pathogens to their hosts by passing through the skin during the feeding process. Their abundance and activity are influenced by abiotic factors including climate, and biotic factors such as host community composition and the presence of predators and competitors [2,5,12]. Forest ecosystems favour the abundance of Acari vectors because of the favourable microclimate and potential vertebrate hosts. A meta-analysis by Bourdin et al. [13] found that *Ixodes* ticks were on average more abundant and taxonomically more diverse in forests than in any other non-forested habitats. A systematic review by Elliott et al. [14] identified the following suitable habitats for chiggers as “artificial wasteland as a result of rural abandoned clearings due to shifting cultivation practices, domestic or suburban neglected areas or neglected gardens and plantations; water meadows including the grassy edges of water bodies and seepages in drier areas; and hedgerows or fringe habitats, typically where two types of habitat meet such as forest edges (ecotones)” based on the early study by Audy and Harrison [15]. More recent research included in the systematic review enabled Elliott et al. [14] to further emphasize the role of ecotones between forest and shrubland areas.

Forests, woodlands and more extensive tree covers are dynamic ecosystems undergoing constant changes, whether due to anthropogenic factors or natural processes, such as loss or expansion [16]. Changes in ecosystem structure and function can modify habitat-host-vector-pathogen interactions. Forest characteristics directly affect the microclimate conditions, which, in turn, may influence the presence and survival of Acari vectors. Additionally, the abundance and composition of host communities, on which Acari vectors feed and potentially transmit pathogens, are influenced by the resources and habitat provided by the forest.

Forest cover dynamics, by modifying the microclimate conditions and the host species composition, may lead to complex, often context-dependent and non-predictable risks of Acari-borne diseases for humans, domestic animals, and wildlife [17]. As a global emerging problem, these effects have been locally addressed in several parts of the world in recent years. However, to our knowledge, all this information has never been gathered and summarized to produce a comprehensive overview of the links between forest cover dynamics, Acari vectors and Acari-borne diseases.

In this study, we present the results of a scoping review, which aims to explore how studies have investigated the links between forest cover dynamics and the transmission of Acari-borne diseases worldwide. More precisely the questions of this article are: (a) What data were used and how were they used to characterise the link between forest cover dynamics and Acari-borne disease risk? (b) What are the major effects induced by forest factors? (c) What are the opportunities, challenges and key areas for further research?

To answer these questions, we described the type of pathogens, vectors, wildlife and geographic range under examination using the PRISMA-SCR method [18]. Then, we reported the methodologies used to describe forest cover dynamics. We quantified the effects of forest cover dynamics on Acari-borne disease risk to identify knowledge gaps for future studies.

## 2 Material and methods

### 2.1 Protocol

Our protocol was drafted following the guidance of Arksey and O'Malley [19] and was guided by the PRISMA extension for scoping reviews which provides a useful checklist of essential items in a scoping review.

### 2.2 Eligibility criteria

All articles included in the scoping review were selected regardless of the study design, articles could be empirical studies, meta-analysis, book chapter or literature review. No restrictions were applied neither to the study geographical location or on the time span. Peer reviewed journal articles were included if they were written in English and dealt with evolving forest or forested area in a context of Acari-borne zoonotic diseases. To ensure these selection criteria were met, a key word count was conducted on the entire article when the reading of the abstract was not sufficient. If the occurrence of “forest” or synonyms was above 10, then the article was selected, otherwise each instance of the word “forest” was considered. If it was a context element or a word from the bibliography, the article was discarded. However, if the forest was part of a study result, the article was retained. Names describing forest cover dynamics had to be mentioned by the authors to be eligible and classified. Additionally, all kinds of methodologies to address these dynamics were included, which might encompass the comparison of static cover. Every article that dealt with non-Acari vectors like mosquitoes, was excluded. Table 1 showed a summary table of the eligibility criteria.

**Table 1:** Summary of the eligibility criteria used for the scoping review.

**Criteria**	**Decision**
Any study design or methodology	Inclusion
Any geographical area	Inclusion
Any publication date	Inclusion
Forest or woodland specifically addressing land cover changes or spatiotemporal evolution.	Inclusion
Acari-borne zoonotic diseases	Inclusion
Not addressing forest cover dynamics as the main subject or as a factor	Exclusion
Not addressing vector-borne zoonotic diseases	Exclusion
Vector-borne diseases but not transmitted by Acari vectors	Exclusion
Not written in English	Exclusion

### 2.3 Information sources

To retrieve all relevant articles, three main databases were searched without time limits: PubMed, Scopus and Web of Science. The search strategies were tested and approved by all co-authors. After collecting all references from the three databases, a complementary and simplified search on Google Scholar was performed.

### 2.4 Search

A preliminary literature review was conducted to effectively design the search strategy (see Additional file 1: Text S1). Following Arksey and O'Malley's framework [19], which corresponds to the first stage of identifying the resFearch question. Through this process, a list of forest cover dynamics relevant to the study of Acari-borne diseases was compiled to be used in the search keywords. The preliminary literature examination (described in the additional file 1: Text S1) categorized forest cover dynamics into four distinct types. Firstly, "deforestation" entails the loss of forest cover whether of anthropogenic origin or not [20]. Secondly, "fragmentation" refers to the conversion of formerly continuous forest into patches of forest separated by non-forest lands [21]. Thirdly, we considered a category not explicitly defined by the FAO but recognized in scientific discourse as "conversion". We defined "conversion" as the transition from spontaneous forests to commercially oriented woody plantations. Finally, “reforestation”, as defined by the FAO [20], is the re-establishment of forest through planting and/or deliberate seeding on land classified as forest, which excludes natural regeneration. We broadened the scope of "reforestation" to encompass the establishment of forests through planting, deliberate seeding or natural regeneration on land previously classified as forest or not. This expanded definition differs from the one originally provided by the FAO [20], as it now combines reforestation, afforestation, and natural regeneration. We have adopted this approach to address the scarcity of information available on the examined forests in the literature.

Three parts composed the final search strategy. The first part is based on all the synonyms for forest. The second part qualified the vectors and diseases, combining broad epidemiological terms with specific vocabulary commonly found in Acari-borne disease research [6]. The third part described all the forest cover dynamics based on a preliminary literature review described in the additional file 1: Text S1. The final query is:

(forest OR “forest ecosystem” OR “forest cover” OR “wooded area” OR woodland OR jungle OR wilderness) AND

("vector-borne disease” OR “tick-borne disease” OR “lyme disease” OR “scrub typhus” OR “disease risk” OR “enzootic hazard” OR “mite-borne disease” OR “arthropod-borne disease”) AND

(deforestation OR fragmentation OR reforestation OR afforestation OR “woodland expansion” OR “woodland encroachment” OR “woodland modification” OR “woodland degradation” OR “tree plantation” OR conversion)

This search strategy was performed at the title and abstract levels on the May 15, 2023.

After going through the retrieved documents, the articles were mainly composed of tick-borne diseases studies. The preliminary literature review highlighted that non-tick Acari-borne diseases constituted a much more limited field of research. A second complementary search was performed using Google Scholar to ensure that all the relevant materials were retrieved. Google Scholar is a powerful search engine to retrieve rarer materials. However, the advanced query has limited character number allowance, leading us to adapt the search query. The complementary query was written as follows:

(forest OR “forest ecosystem” OR “forest cover” OR “wooded area” OR woodland OR jungle OR wilderness) AND (“scrub typhus” OR “mite-borne disease” OR “Acari-borne disease” OR “orientia tsutsugamushi”) AND (deforestation OR fragmentation OR reforestation OR afforestation OR “woodland expansion” OR “woodland encroachment” OR “woodland modification” OR “woodland degradation” OR “tree plantation” OR conversion)

This search was performed in all fields on the September 7, 2023.

### 2.5 Selection of sources of evidence

Before selecting articles, the four authors amended and validated the search query, selection criteria, and the data extraction methodology. One author was responsible for all the steps of articles screening, going from the selection based on the evaluation of titles, the evaluation of abstracts, to the final full text evaluation. Meanwhile, she performed the data extraction. To check validity of the selection and data extraction, one additional author randomly screened a smaller sample of articles and independently charted the data. Any possible disagreements on article selection or data extraction were resolved by consensus.

### 2.6 Data extraction

The data-charting form was developed based on the preliminary literature review and validated by all four authors. This form is accessible in the additional file 1: Text S2. We used Zotero [22] and spreadsheet to retrieve extracted data from the articles. A variable dictionary is available in the additional file 2: Dataset S3. Concerning the data items, as mentioned by Arksey and O'Malley [19], we retrieved data on i) the article characteristics (such as the authors, year of publication, location studied etc), ii) the objects of study (information on disease, vector, hosts, humans, forest cover dynamics), iii) the objectives (research question, gaps, variables to be explained), iv) the methodology (type of study, methodology, forest parameters, statistical analysis, spatial and temporal scales, indicators used, methods to describe the dynamics) and v) the results (important summarized results, qualitative classification of the effects of the forest, limitations described by the authors). Information concerning the research question, the methods, a brief summary of the results and the limitations was retrieved as explanatory texts for the narrative and qualitative analysis. The rest was directly translated into qualitative or quantitative variables to further perform descriptive statistical analysis such as the estimation of proportions, or simple indicators of dispersion (median, interquartile distance, range). Some classifications were created *ad hoc* while screening the articles, such as the forest parameters or the methods to describe the forest temporal dynamics.

### 2.7 Synthesis of the results

We synthesized the findings through a narrative approach. First, we categorized articles based on their exploration of forest cover dynamics and Acari-borne diseases. Then, we delineated the various pathogen types, vectors, hosts and humans investigated. This was followed by a detailed account of forest characteristics. In this part, qualitative classifications were made post hoc after data extraction based on what was found in the selected articles. In evaluating the impact of forest cover dynamics, we organized studies according to the specific dynamics under investigation, such as fragmentation, deforestation, conversion, or reforestation. After an overall presentation of the articles, we presented the results for the empirical or modelling articles (n = 86) separately from the literature synthesis and review articles (n = 25) encompassing the literature review, meta-analysis, expert opinion papers and popular science. We split the two categories to control for bias associated with possible duplication of information.

## 3 Results

### 3.1 Overview of selected studies on acari-borne diseases and forest cover dynamics

**Fig. 1:**
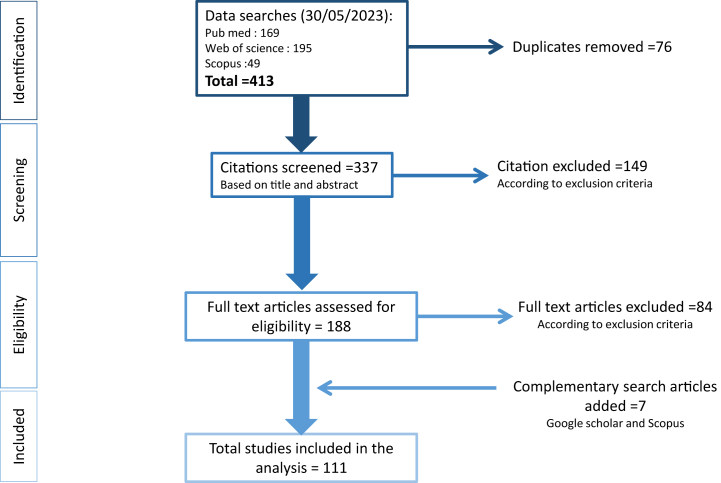
Flow diagram for scoping review of forest cover dynamics impacts on Acari-borne disease literature. This flow diagram is based on PRISMA-ScR methodology.

**Fig. 2:**
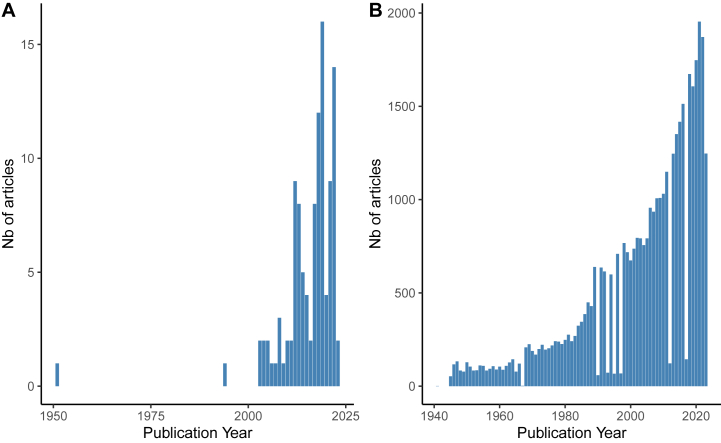
Chronological charts depicting the publication periods of the articles. (A) represents the articles collected thanks to the request and included in the scoping review and (B) the articles extracted from PubMed with the broad request “tick-borne disease” as an element of comparison.

The search strategy identified 413 articles from the three databases. After removing the duplicates, 337 abstracts and titles were screened and 188 of them were selected for a full-text review. Seven articles from the complementary search were added. A total of 111 relevant articles were selected for the scoping review (see Fig. 1). The complete list of those articles in the analysis grid is available in the additional file 2: Dataset S2. Among the 111 articles analysed in this review, 70 % were empirical studies (n = 78), while literature reviews made up 15 % of the total (n = 17). The remaining articles included a smaller proportion of meta-analyses, modelling papers, science popularization pieces, and expert opinion contributions (see Table 2). Fig. 2 A reveals an increasing interest in tick-borne diseases since the 1970s. It was not until the early 2000s that most authors started addressing forest cover dynamics and Acari-borne diseases together (see Fig. 2 B).

**Table 2:** Type of collected articles.

	**Overall (N = 111)**
**Nature**	
Empirical studies	78 (70.3 %)
Expert opinion	2 (1.8 %)
Literature Review	17 (15.3 %)
Meta-analyse	3 (2.7 %)
Modelling	8 (7.2 %)
Science popularization	3 (2.7 %)

Most of the research exploring the relationship between Acari-borne diseases and forest cover dynamics focused on tick models and tick-borne diseases. Out of the 111 articles, 53 focused mostly on *Ixodes* and Lyme disease in North America and 27 in Europe. Only 12 articles focus on Asia and even fewer on the rest of the world (see Table 3).

**Table 3:** Number and percentage of articles sorted by relevant categories.

	**Empirical-Model Breakthrough (N = 86)**	**Literature Synthesis (N = 25)**	**Overall (N = 111)**
**Continent**			
Africa	1 (1.2 %)	0 (0 %)	1 (0.9 %)
Asia	10 (11.6 %)	2 (8.0 %)	12 (10.8 %)
Europe	25 (29.1 %)	2 (8.0 %)	27 (24.3 %)
North America	46 (53.5 %)	7 (28.0 %)	53 (47.7 %)
South America	3 (3.5 %)	1 (4.0 %)	4 (3.6 %)
Worldwide	1 (1.2 %)	12 (48.0 %)	13 (11.7 %)
Oceania	0 (0 %)	1 (4.0 %)	1 (0.9 %)
**Forest**			
Both	1 (1.2 %)	7 (28.0 %)	8 (7.2 %)
Temperate	72 (83.7 %)	14 (56.0 %)	86 (77.5 %)
Tropical	13 (15.1 %)	4 (16.0 %)	17 (15.3 %)
**Vector**			
Both	1 (1.2 %)	1 (4.0 %)	2 (1.8 %)
Chigger	4 (4.7 %)	1 (4.0 %)	5 (4.5 %)
Tick	80 (93.0 %)	17 (68.0 %)	97 (87.4 %)
Missing	1 (1.2 %)	6 (24.0 %)	7 (6.3 %)

As seen in Table 4, out of the 11 identified pathogens in the reviews, eight were bacteria. The number of pathogens studied varied according to the type of forest. In temperate forests, 72.5 % of the examined pathogens were *Borrelia* spp. responsible for Lyme disease, followed by *Anaplasma* spp. responsible for anaplasmosis (11.8 %). In tropical forests, the scientific community equally prioritized Kyasanur Forest Disease Virus, *Orientia tsustugamushi* responsible for scrub typhus and *Rickettsia* spp. (resp. 22.7 %, 22.7 % and 18.2 %).

**Table 4:** Distribution of examined pathogens in the dataset.

	Empirical-Model Breakthrough		Literature Synthesis		Overall^a^	
	Temperate (N = 87)	Tropical (N = 14)	Temperate (N = 15)	Tropical (N = 8)	Temperate (N = 102)	Tropical (N = 22)
Pathogen						
*Borrelia* (bacteria)	61 (70.1 %)	0 (0 %)	13 (86.7 %)	1 (12.5 %)	74 (72.5 %)	1 (4.5 %)
*Anaplasma* (bacteria)	12 (13.8 %)	1 (7.1 %)	0 (0 %)	1 (12.5 %)	12 (11.8 %)	2 (9.1 %)
*Rickettsia* (bacteria)	4 (4.6 %)	1 (7.1 %)	0 (0 %)	3 (37.5 %)	4 (3.9 %)	4 (18.2 %)
*Babesia* (protist)	5 (5.7 %)	0 (0 %)	1 (6.7 %)	0 (0 %)	6 (5.9 %)	0 (0 %)
*Ehrlichia* (bacteria)	4 (4.6 %)	0 (0 %)	1 (6.7 %)	1 (12.5 %)	5 (4.9 %)	1 (4.5 %)
*Orientia* (bacteria)	0 (0 %)	3 (21.4 %)	0 (0 %)	2 (25.0 %)	0 (0 %)	5 (22.7 %)
KFDV^b^ (virus)	0 (0 %)	5 (35.7 %)	0 (0 %)	0 (0 %)	0 (0 %)	5 (22.7 %)
*Bartonella* (bacteria)	0 (0 %)	2 (14.3 %)	0 (0 %)	0 (0 %)	0 (0 %)	2 (9.1 %)
*Coxiella* (bacteria)	0 (0 %)	1 (7.1 %)	0 (0 %)	0 (0 %)	0 (0 %)	1 (4.5 %)
*Francisella* (bacteria)	0 (0 %)	1 (7.1 %)	0 (0 %)	0 (0 %)	0 (0 %)	1 (4.5 %)
LIV^c^ (virus)	1 (1.1 %)	0 (0 %)	0 (0 %)	0 (0 %)	1 (1.0 %)	0 (0 %)

a As on article can deal with several type of pathogen, the overall amounts to 122.

b KFDV=Kyasanur Forest Disease Virus.

c LIV = Looping Ill Virus.

### 3.2 Addressing pathogen, vector, host and human roles in the context of acari-borne disease transmission

In empirical and modelling studies, the methodologies addressing the connection between forests and Acari-borne diseases appeared heterogeneous and mainly focused on the pathogen. Of all studies, 22.1 % investigated pathogens and humans, 16.3 % looked at pathogens, vectors and wildlife, 12.8 % considered only pathogens and wildlife, and 10.5 % looked at the vectors and the wildlife without the pathogens. Finally, 21 % of the studies did not consider pathogen presence focusing on vectors as a proxy for estimating the hazard of disease transmission and 1.8 % used wildlife as a proxy (see the Venn diagrams in the additional file 3: Fig. S4 for the empirical and review articles distributions). Ticks were investigated in 80 empirical or modelling articles, while chiggers were the subject of only four articles and one article considered both (see Table 3).

**Fig. 3:**
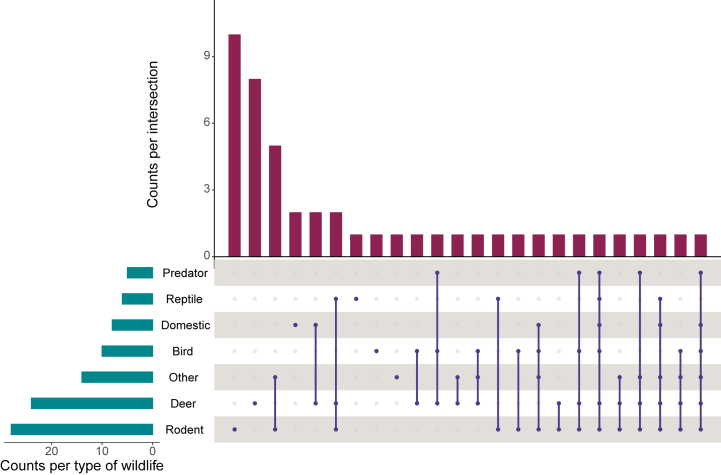
Upset plot of the studied wildlife distribution for empirical or modelling studies only (n = 47). The upper chart represents the number of articles in y axis according to the pattern of studied wildlife in x axis. For example, 10 articles only examine rodents population, eight deal with deer only, while 5 of them consider both rodents and other small and medium mammals. The chart on the left-hand side, represent the overall number of articles by wildlife types. For example, in total, rodents are investigated in 28 empirical articles, and reptiles only in six articles.

Regarding the studied wildlife, we identified the presence of deer, rodents, mammal carnivores, domestic animals (dogs, cats, cattle, etc.), birds, other mammals (excluding rodents, domestic animals and predators) and reptiles across empirical and modelling articles (see Fig. 3). The primary emphasis was on rodents (n = 28), deer (n = 24), and other mammals (n = 14). Specifically, 10 studies focused only on rodents followed by those exclusively examining deer (n = 8), which are respectively the most important hosts for *Ixodes* ticks and the main reservoirs for *Borrelia burgdorferi* [23]. Twenty-eight empirical or modelling articles also assessed the wildlife that may not directly serve as reservoirs by using camera trapping, capture, mark-recapture or mark-resight, collaborating with veterinarians or hunters or using biodiversity databases. Five studies investigated the role of cattle grazing, as cattle may not be a pathogen reservoir but a host of interest. Each study found that pasture alone was not a suitable habitat for the vector to thrive. However, combining forest encroachment or fragmentation with pasture created a landscape that increased tick abundance and pathogen prevalence. This effect was observed in both tropical and temperate contexts. Livestock abundance or the time they spent in the pasture was significant in only two studies [24,25]. Four empirical or modelling articles considered carnivores, but they were viewed as hosts or for their role in vector dispersion, rather than for their regulatory capacity. The distribution of studied wildlife in literature synthesis and review articles is available in the additional file 3, Fig. S5.

Humans have a particular place in the system because they are hosts for the pathogens, and they also have an important impact on forest cover dynamics as users or through their governance. Of the 86 empirical or modelling articles, 40.7 % involved humans in some capacity, such as hosts infected with the pathogen, infested with the vectors, forest users, or participants in governance and management interventions. Most articles (25.6 %, n = 22) investigated pathogen prevalence in humans, viewing them as a potentially vulnerable population. This perspective had implications for public health. Ten articles (11.6 %) considered humans as users meaning they considered their activities or behaviour in forests and their impacts. Five articles (5.8 %) dealt with governance, meaning policies, conservation practices or institutions regulating forests and forest resources. In contrast, literature synthesis articles put more emphasis on humans with 21 out of 25 articles dealing with humans. They were mainly considered as users (40 %) followed by governance and vulnerable population (resp. 24 % and 20 %).

### 3.3 Exploring forest cover dynamics in the context of acari-borne disease transmission

**Fig. 4:**
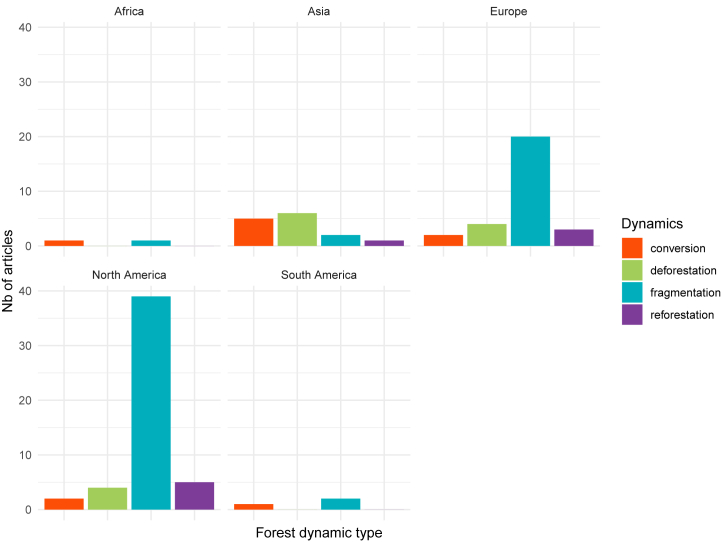
Distribution of forest cover dynamics studied according to the continent, for georeferenced empirical and modelling studies (n = 85).

Articles either focused on one specific forest cover dynamic or studied two or several together. Fragmentation is the most frequently studied dynamic regarding its impact on Acari-borne disease transmission. Out of the 86 empirical or modelling articles, 17 % of them focused on deforestation, 74 % on fragmentation, 14 % on forest conversion and 12 % on reforestation. Fig. 4 illustrates the diversity of studied forest cover dynamics according to the continent under examination for empirical and modelling georeferenced studies. The effect of deforestation was mostly investigated in tropical areas, constituting 50 % of tropical forest empirical or modelling studies compared to 12 % of temperate forests studies. Conversion was overrepresented in tropical areas as well, with 50 % of tropical forests studies, whereas temperate area accounted for 8 % of temperate forests studies. Conversely, fragmentation was predominantly investigated in temperate forest studies, comprising 81 %, in contrast to 36 % in tropical areas. Reforestation was underrepresented in both contexts with 12 % of the studies located in temperate areas and 14 % in tropical areas.

A diverse array of variables was used to describe forest cover dynamics. We listed them and we identified in which forest cover dynamic category they best fit into. Table 5 retrieves all the forest variables used according to their spatial and temporal scales. Common factors included surface area and forest type (deciduous, coniferous, or mixed). Additional indices encompassed configuration elements such as the number, surface, connectivity, aggregation, density, shape, and length of patches, as well as edge characteristics and neighbourhood features. Some authors analysed composition parameters, including species composition, diversity of plant species, litter and soil composition. Forest structure parameters, such as stand story, tree age, biomass, height, diameter at breast height, canopy cover, and dead wood percentage were also considered. Functionality indicators, such as food resource availability for hosts or habitat provisions, were examined in two studies. Finally, some articles mentioned the type of forest management as a parameter. The number of parameters varied widely across empirical or modelling articles, ranging from 0 to 14 (median = 3) and an important variability (with an inter-quartile distance of 3). Empirical or modelling studies on fragmentation dynamics employed the highest number of variables (median = 4) compared to deforestation, conversion and reforestation that all had a median of 2. Configuration variables were the most commonly used variables for each forest cover dynamic, but we found that they most often described fragmentation and deforestation dynamics. Composition as well as structure variables were most commonly used to describe reforestation and conversion dynamics. Functionality variables were only used in two studies on fragmentation and human factors were considered for both reforestation and conversion dynamics. Certain groups of indicators were better suited for different spatial scales, ranging from patch to landscape, to country and to global. As seen in Table 5, the patch scale was well adapted to local species or litter composition. Metrics of forest structure were also more suitable because they could be measured directly on the sample plot. At the landscape scale, the authors chose forest configuration indices that were directly estimated from a landcover map. Thus, the choice of forest variables was determined by the data availability, the research question and the related spatial scale.

**Table 5:** Classification of forest variables used according to spatial and temporal scale in the collected articles. The variables are classified by types (composition, configuration, structure, functionality, human factors) for each dimension. In rows, the temporal scale is indicated in years. (0–5 = the authors monitored the forest variables for a period of time extending for zero to five years, 6–10 = for six to 10 years, 11–99 = for 11–99 years, 100+ = for more than 100 years). In columns, the spatial scale representing the extent of area where authors had considered the ecological process. They had been classified in plot or patch, landscape, region or country and more. For a description of each forest variable see Additional file 4: Dataset S7.

Spatial scale	Plot/Patch	Landscape	Region	Country +
Temporal scale				
0–5	**Composition:**	**Composition:**	**Composition:**	**Composition:**
	Species	Species (Categorical)^a^	Species (Categorical)^a^	Species (Categorical)^a^
	Soil	Soil	Soil	Wildlife
	Invasive species	Diversity	Diversity	
	Wildlife		Wildlife	
	Diversity			
	Litter			
	**Structure:**	**Structure:**	**Structure:**	**Structure:**
	Density	Density		
	Canopy			
	Stories			
	Mortality (coarse-woody debris)			
	Height and DBH			
	Age			
	Biomass			
	**Configuration:**	**Configuration:**	**Configuration:**	**Configuration:**
	Area	Area	Area	Area
	Edge/perimeter	Edge/perimeter	Edge/perimeter	Edge/perimeter
	Neighbour	Number of patches	Number of patches	
	Shape	Neighbour	Neighbour	
		Connectivity	Connectivity	
		Aggregation	Aggregation	
		Shape	Shape	
	**Functionality:**	**Functionality:**	**Functionality:**	**Functionality:**
	Food resources			
	Habitat resources			
	**Human factor:**	**Human factor:**	**Human factor:**	**Human factor:**
	Management			
6–10	**Composition:**	**Composition:**	**Composition:**	**Composition:**
	Species (Categorical)^a^	Species (Categorical)^a^	Species (Categorical)^a^	Species (Categorical)^a^
	**Configuration:**	**Configuration:**	**Configuration:**	**Configuration:**
	Edge/perimeter	Edge/perimeter	Edge/perimeter	Edge/perimeter
	Area	Area	Area	Area
	Neighbour	Number of patches	Number of patches	
11–99	**Composition:**	**Composition:**	**Composition:**	**Composition:**
			Species (Categorical)^a^	Species (Categorical)^a^
	Species (Categorical)^a^	Species (Categorical)^a^	Wildlife	Wildlife
	Diversity	Diversity	Diversity	Diversity
	**Structure:**	**Structure:**	**Structure:**	**Structure:**
	Canopy			
	Stories			
	Age			
	**Configuration:**	**Configuration:**	**Configuration:**	**Configuration:**
	Area	Area	Area	Area
	Neighbour	Neighbour	Edge/perimeter	Edge/perimeter
	Edge/perimeter	Number of patches	Neighbour	
		Edge/perimeter	Number of patches	
100+	**Composition:**	**Composition:**	**Composition:**	**Composition:**
		Species (Categorical)^a^		
	Species (Categorical)^a^	Wildlife	Species (Categorical)^a^	Species (Categorical)^a^
	Invasive species	Invasive species	Wildlife	Wildlife
	**Structure:**	**Structure:**	**Structure:**	**Structure:**
	Canopy			
	Stories			
	**Configuration:**	**Configuration:**	**Configuration:**	**Configuration:**
	Area	Area	Area	Area

a Categorical = broad category of the forest nature (dominance of deciduous, coniferous or mix species).

Empirical or modelling studies almost equally investigated forests on both large and small dimensions. On the large dimension (47 %), studies covered several tens to hundreds of kilometres or more examining regions, countries, or continents. On the small dimension (45 %), studies encompassed several kilometres corresponding to villages or isolated sample plots. Additionally, 8 % of empirical or modelling studies considered both small and large dimensions, transitioning between the two.

The temporal scale poses a real challenge, as forest ecological processes may not align with the scientific temporality. Hence the authors of empirical or modelling studies have developed various methodologies to adapt temporal scale and data availability for characterising forest cover dynamics. We listed five of them. First, in 12 studies, the authors used two different maps with a determined time difference to compute rates of change (deforestation, reforestation). Second, in three studies, the authors reconstructed a chronosequence in the field to determine the amount of time between the oldest and the newest forests comprising the chronosequence. Third, in 67 articles, a proxy was computed using a map (edge, perimeter, perforation, aggregation etc …) or in the field. This method was largely used for fragmentation to estimate and compare forest fragmentation levels. Fourth, when the perturbation was short term and adapted to small spatial dimensions, the authors could measure forest parameters directly in the field during the ecological sampling (vectors or hosts). This was the case for two articles, both with a 3-year sampling period. Fifth, in one article, the authors developed a predictive model of the forest occupancy based on climatic data.

**Fig. 5:**
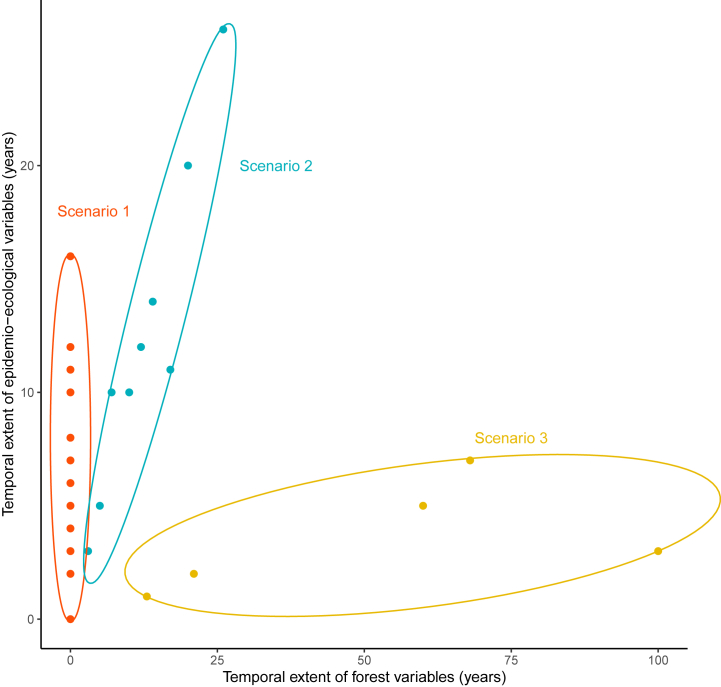
Temporal extent of eco-epidemiological data as a function of temporal extend cover by forest data. Three scenarios emerged from this chart. Scenario 1 encompasses studies incorporating both short and long-term epidemiological-ecological data alongside current forest data. Scenario 2 comprises studies aligning the temporal scope of their epidemiological-ecological data with that of their forest data. Scenario 3 gathers studies with long-term forest data but shorter-term epidemiological-ecological data. This classification is established based on the insights provided by the chart.

Despite concerted efforts, reconciling eco-epidemiology and forest temporal scales remained challenging. Three scenarios emerged (see Fig. 5). Some authors (n = 67) used ecological or epidemiological data covering from 0 to numerous years and viewed the forest as a snapshot, particularly evident in studies investigating fragmentation. This case was represented in Fig. 5 by scenario 1. A few authors (n = 5) captured extensive periods of forest change using temporal maps but lacking ancient ecological data (scenario 3). Lastly, 14 articles managed to match their eco-epidemiological data with the temporal changes in the forest parameters (scenario 2).

### 3.4 Understanding the complex effects of forest change on pathogen and vector presence

A comprehensive analysis revealed a lack of consensus on the outcomes of reforestation, deforestation, forests fragmentation or conversion on pathogens and vectors dynamics. Five types of effects have been identified in the literature. An increase in forest surface could increase or decrease the pathogen or vector presence. The relationship between the two can be non-linear or non-significant. Lastly, the authors can identify dependent effects that alter the relationship.

For all combined forest cover dynamics, the authors of empirical or modelling articles equally observed a protective and a risk effect of the forest on the pathogen (prevalence, incidence, etc). Increasing the forest surface area would decrease or increase the presence of the pathogen in 20.9 % of the articles, respectively (see Table 6). Regarding the effect of forest on the vector presence, forest surface tended to have a main risk effect observed in 19.8 % of the empirical or modelling articles. However, this percentage remained quite low and close to the protective effect (see Table 7).

**Table 6:** Distribution of articles by effects on pathogens presence, forest cover dynamics, spatial and temporal dimensions. Table 6 classifies the different effects of forest on the pathogen presence according to the type of forest cover dynamic, the spatial scale used in the articles (represented by the categories large, small or both) and the temporal scale used in the articles (represented by the median and interquartile of the temporal extend for which forest variables have been retrieved).

Type of effects		Number of articles	Nb. of articles by forest cover dynamics	Nb. of articles by spatial dimensions	Median and interquartile of forest temporal scale variables^a^
All articles		89			
	*Forest surface increases pathogens*	18 (20.9 %)	Deforestation: 2	Large:7	Median: 0
			Fragmentation:14	Small:11	Interquartile:0
			Conversion:2	Both:0.	
			Reforestation:2		
	*Forest surface decreases pathogens*	18 (20.9 %)	Deforestation:5	Large:10	Median: 0
			Fragmentation:12	Small:6	Interquartile: 10
			Conversion:2	Both:2	
			Reforestation:1		
	*Non-linear relationship*	3 (3.5 %)	Deforestation:1	Large:2	Median: 0
			Fragmentation:2	Small:0	Interquartile: 10
			Conversion:1	Both:1	
			Reforestation:1		
	*Non-significant relationship*	8 (9.3 %)	Deforestation:0	Large:4	Median: 0
			Fragmentation:7	Small:4	Interquartile: 0
			Conversion:1	Both:0	
			Reforestation:1		
	*Dependant effects*	15 (17.4 %)	Deforestation:2	Large:9	Median: 0
			Fragmentation:14	Small:4	Interquartile: 0
			Conversion:1	Both:2	
			Reforestation:1		
	*Non-studied*	24 (27.9 %)	Deforestation:5	Large:8	Median: 0
			Fragmentation:15	Small:14	Interquartile:0
			Conversion:5	Both:2	
			Reforestation:4		

a In Years.

**Table 7:** Distribution of articles by effects on vectors presence, forest cover dynamics, spatial and temporal dimensions. Table 7 classifies the different effects of forest on the vector presence according to the type of forest cover dynamic, the spatial scale used in the articles (represented by the categories large, small or both) and the temporal scale used in the articles (represented by the median and interquartile of the temporal extend for which forest variables have been retrieved).

Type of effects		Number of articles (%)	Nb. of articles by forest cover dynamics	Nb. of articles by spatial dimensions	Median and interquartile of forest temporal scale variables^a^
All articles		89			
	*Forest surface increases vector*	17 (19.8 %)	Deforestation: 4	Large: 5	Median: 0
			Fragmentation:11	Small:12	Interquartile:0
			Conversion:2	Both:0	
			Reforestation:4		
	*Forest surface decreases vector*	10 (11.6 %)	Deforestation: 3	Large:6	Median: 0
			Fragmentation: 7	Small:3	Interquartile:0
			Conversion:1	Both:1	
			Reforestation:1		
	*Non-linear relationship*	2 (2.3 %)	Deforestation: 1	Large:1	Median: 0
			Fragmentation: 1	Small:1	Interquartile:0
			Conversion: 0	Both:0	
			Reforestation: 1		
	*Non-significant relationship*	5 (5.8 %)	Deforestation: 0	Large:3	Median: 0
			Fragmentation:4	Small:2	Interquartile:0
			Conversion:1	Both:0	
			Reforestation:0		
	*Dependant effects*	8 (9.3 %)	Deforestation: 1	Large:0	Median: 0
			Fragmentation: 5	Small:5	Interquartile:0,75
			Conversion: 3	Both:3	
			Reforestation: 0		
	*Non studied*	44 (51.2 %)	Deforestation:6	Large:25	Median: 0
			Fragmentation: 36	Small:16	Interquartile:0
			Conversion: 5	Both:3	
			Reforestation: 4		

a In Years.

**Fig. 6:**
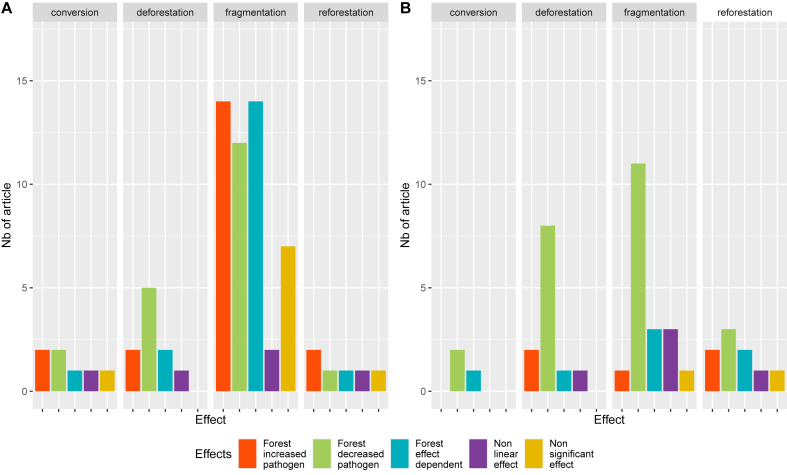
Differences in forest cover dynamics impacts distribution on the pathogen presence according to studies' type. (A) represents the distribution when considering only empirical and modelling articles (n = 86) and (B) is the distribution when considering only review studies based on existing literature n = 25.

Different trends were observed for forest cover dynamics when comparing the entire sample of papers to a subset comprising only literature synthesis articles. Fig. 6 shows an illustration of the pathogen presence. In a context of fragmentation, more empirical and modelling articles found either a risk or a dependent effect of the forest (see Fig. 6 A) compared to what is reported in literature synthesis and review articles (see Fig. 6B). The same trend applied to the vector presence (See additional file 3: Fig. S6). The limited availability of studies addressing reforestation and conversion makes it difficult to draw conclusions on their impact on disease transmission.

Dependent effects were identified across various empirical and modelling studies, with authors acknowledging and considering these dependencies in their analyses. A dependent effect is a factor that modifies how forest cover dynamics influence disease risk. Such factors were related to spatial scale and the buffer size, the vector species or stages, the pathogen species, the location sites and the biogeographic region, the neighbouring effects, the structure or configuration of forest, the host composition or the proportion of exposed humans.

**Fig. 7:**
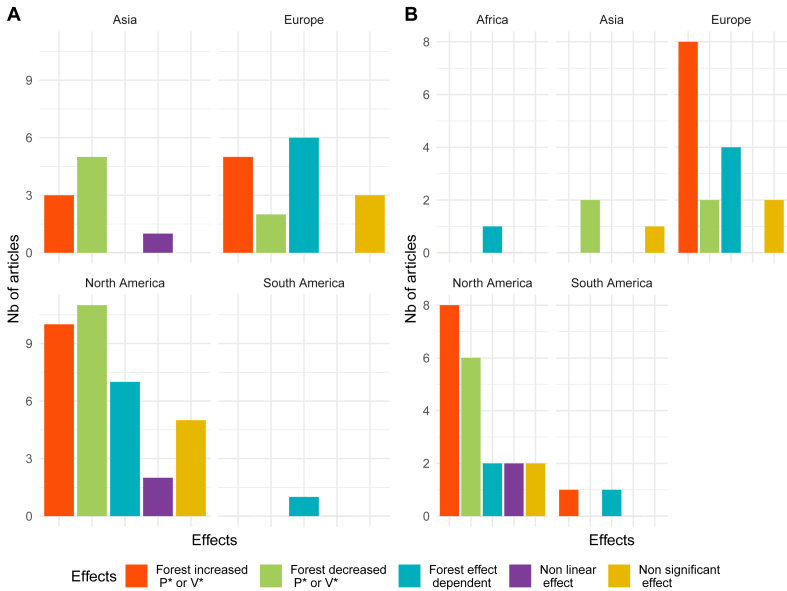
Distribution of the observed forest effects according to the continent, for georeferenced empirical and modelling studies (n = 85). (A) chart represents the impact of forest cover size on the pathogen presence (∗P meaning pathogen) and (B) chart on the vector presence (∗V meaning vector).

Apart from the forest cover dynamics, impacts on pathogen and vector presence also depended on the continent of the studied sites of empirical articles (see Fig. 7 A for articles studying the pathogen and Fig. 7 B for articles studying the vector). Proportionally, forests were found to be more effective in reducing the risk in North America and Asia than in Europe. It was difficult to assess South America, Africa and Oceania given the low number of studies.

## 4 Discussion

The purpose of this scoping review was to provide an overview of the literature on the links between forest cover dynamics and Acari-borne disease risk. More precisely this review collated all pathogens, vectors, hosts and forest variables. It described how these variables were used and what were the major observed impacts of forest cover dynamics on Acari-borne disease risk worldwide.

The literature is mainly composed of empirical studies producing observational data, highlighting the emerging nature of the studied field. The temperate forests of North America were the most studied followed by European temperate forests and tropical forests of Southeast Asia. The influence of forest cover dynamics on Acari-borne diseases remained a primarily Western-centric concern. Among Acari vectors living in the forest, *Ixodes* ticks transmitting Lyme disease in a context of temperate forest fragmentation were the most investigated. The search query was focused on diseases, so most of the studies found were centred on pathogens. However, few studies used vector density as an indicator for estimating risk. Humans were not systematically considered even though they are a key element of the system and essential to estimating exposure and risk. Variables used to describe forest ecosystems varied considerably and the temporal aspect was challenging to consider. We identified four major theoretical backgrounds from the collected articles as habitat suitability [26,27], island biogeography theory [28], community composition ecology with the dilution effect hypothesis [29] and landscape ecology with ecotone [30] centred studies. The influence of forest cover dynamics on the risk of Acari-borne disease transmission is still debated within the scientific community, reflecting a lack of consensus in terms of observed effects, methodologies and theoretical frameworks used to investigate a complex relationship.

### 4.1 Assessing the link between forest cover dynamics and disease risk: insights from disease ecology theories

According to habitat suitability theories and niche modelling studies, forests and forested areas serve as habitats for many Acari vectors studies (see Ref. [13] for a meta-analysis). Forest ecosystems provide a stable microhabitat required for the vector's survival and support diversified vertebrate communities that serve as hosts for vectors and reservoirs for pathogens. The absence of forests should result in the absence of vectors and a decreasing risk of disease transmission. These statements are supported in several articles examining either vector presence [24,31,32] or vectors and pathogens combined [33] in all forest cover dynamics considered. Modelling studies performed by Robinson et al. [34] for pathogens and by Li et al. [35] for ticks and pathogens corroborated these empirical findings. However, this statement must be nuanced as several authors also found co-dependent effects for the vector presence [36,37] and for the pathogen presence with the meta-analysis of Walsh et al. [38]. Peri-urban areas should not be overlooked since Piedmonte et al. [39] collected more infected ticks in urban environments than in forests. The importance of these highly populated areas challenges the hypothesis that ticks only thrive in forests. Our scoping review emphasized that forests may protect against disease transmission under specific conditions involving forest configuration factors as shown in the studies of Dong et al. [40] and Wongnak et al. [41].

These results invoke the theory of island biogeography by considering forests and forest fragments as habitats for vectors and pathogen reservoirs, further supported by niche modelling studies. Large and well-connected forest patches should support highly diversified plant and animal communities, which may significantly enhance disease maintenance and transmission. Fragmentation, particularly in temperate forests, has received important attention in this field. Some studies observed a positive correlation between larger and highly connected patches of forest and the abundance of vectors and pathogens [42,43]. However, Brownstein et al. [44] observed a similar correlation for human cases but not for the field-collected ticks, suggesting other factors at stake. Wang et al. [45] also corroborated those findings in a reforestation context observing that forest connectivity alone did not significantly affect the presence of pathogens but became significant when combined with other factors such as host assemblage or the infection status of neighbouring areas. These findings support Reperant's conceptualization of hosts, themselves seen as biogeographic islands for pathogens [46]. Such results were reported in tropical environments by Esser et al. [47] where larger forest fragments were associated with more diverse wildlife communities, consequently supporting larger and more diverse tick communities.

Studies investigating forest cover dynamics under the scope of community compositions help to understand these complex heterogeneous effects. Forest composition and configuration, alongside forest structure, can induce vertebrate community reassembly by modifying ecological niches and food resources [48]. A prevailing hypothesis suggests that only generalist species can easily adapt to perturbations, while specialist species struggle to acclimate to anthropized habitats emerging from forest cover dynamics [49]. Generalist species like rodents and deer are the main reservoirs of pathogens and vectors hosts, playing a major role in Acari-borne disease transmission. In a temperate forest marked by fragmentation resulting from fire, MacDonald et al. [50] observed that small mammal communities were completely reorganised. Concurrently, they found a high level of tick burden following fire perturbation. Consistent with those results, Morand et al. [51] showed that tropical forest cover dynamics such as conversion or reforestation also reshaped the rodent and pathogen community composition according to their level of specialization and synanthropy. Highly synantropic rodents were more associated with fragmented landscapes where highly specialised forest rodents decreased. However, in this study *Orientia tsutsugamushi*, the agent of scrub typhus, decreased with ongoing fragmentation and forest conversion to agricultural land [50]. Razali et al. [1] hypothesized that forest remnants with more disturbed surrounding areas contribute to higher intensity of ectoparasite infections, indicating a condition of stress in animals. Although the authors found a non-significant relationship and this hypothesis could not be verified either by Nadolny and Gaff [52].

Habitat suitability, island biogeography and community composition frameworks are often complementary and used in synergy with the dilution effect hypothesis. The dilution effect is defined as “the net effect of species diversity reducing disease risk by any of a variety of mechanisms” [53]. The dilution effect hypothesis is commonly referred to in disease ecology and particularly in Lyme disease. Many authors attempted to link these ecological mechanisms to forest cover dynamics. The study by Allan et al. [54] demonstrated that in fragmented landscapes, biodiversity tended to be lower, leading to higher pathogen density in vectors. These observations were also consistent in tropical forests such as the case of Kyasanur Forest Disease [55]. Linske et al. [56] found the opposite trend with higher biodiversity in fragmented landscapes significantly associated with higher tick and pathogen presence. LoGiudice et al. [57] also failed to establish a significant relationship between forest patch size and prevalence, or between forest patch size and the population of white-footed mice, despite observing a negative yet non-significant trend. Based on this literature, fragmentation has largely been examined within the framework of the dilution effect and is thought to be used as a proxy for biodiversity. The dilution effect has found consistent support in the literature [58]. However, directly associating habitat disturbance like fragmentation and biodiversity loss may show conflicting results by underestimating confounding factors and failing to accurately represent local community ecology [58,59]. In this trend, our scoping review shows an overrepresentation of the protective effect of the forest in reviews compared to empirical studies.

The ecotone and interface framework offers a nuanced perspective on why fragmentation may not always have detrimental effects on biodiversity but may increase disease risk. These hypotheses suggest that fragmentation may create new niches at the interface between two different habitats. These niches can support high species richness but also greater encounter probability with alternative hosts compared to the interior of large forest patches [56,60]. Goethert and Telford [61] found that ticks from the edge fed on a greater diversity of hosts than those from the thicket and that the prevalence of pathogen was higher at the edge. All previously discussed ecological mechanisms enhance our understanding of the abundance of either the vector or the pathogen, and thus assisting to quantify the hazard. However, the risk of vector-borne disease not only encompasses the hazard but also the exposure and the coping capacity [62]. Exposure reflects the probability of getting potential vector-infected bites. Ecotone and interface resulting from forest cover dynamics, by creating spatial overlap between tick-infested areas and human visits, then increase the exposure and thus the risk [[63], [64], [65], [66]]. Jackson et al. [67] observed that Lyme disease human cases had a quadratic relationship with the percentage of forest cover. However, human cases also significantly increased with the percentage of forest edge, meaning that when forest cover is equivalent, the interspersion between two habitats is of great importance for disease risk. These results were confirmed by McClure and Diuk-Wasser [68] and brought back the forest configuration at the core of the debate. In a context of fragmentation or deforestation, even if an important surface of a niche vanishes, it creates more and more opportunities for exposure between species that do not usually encounter each other or even between wildlife, domestic animals and humans. The results of our scoping review aligned with this hypothesis with the overall positive effect of deforestation on disease risk.

### 4.2 Challenges in assessing forest cover dynamics: spatial and temporal scale interplay in disease risk

The diversity of approaches, theories, hypotheses and the number of context-dependent factors are not sufficient to explain the conflicting results. Studying the impact of forest cover dynamics on disease risk is also challenging because of different temporal and spatial scales intertwining. Concerning the spatial scale, there is a difference between entomological hazard that is usually measured at the patch scale and human exposure that is investigated at a broader scale by the public health surveillance system. Several authors pointed out the dependency with the spatial scale on their results for the pathogen [69,70], for the vector [36,71] or for both [72]. McClure and Diuk-Wasser [68] built a model to connect both. This difference has also been more widely acknowledged by Salkeld et al. [73] in their meta-analysis. Studies that relied on county-level health data were not incorporated for two reasons. First, infection dynamics operate at fine spatial scales and therefore data at coarse county or region levels may not be indicative of local pathogen transmission patterns. Second, sampling effort can be very heterogeneous at the regional level making it harder to obtain an accurate measure of biodiversity. These scaling effects have already been acknowledged in ecology [74,75]. In epidemiology, it is also common to study short ecological processes. Our scoping review showed that very few authors considered the age of the forest in their analyses. Forest cycles run over a very long period compared to the lifetime of humans, wildlife and even more vectors. Shah et al. [66] highlighted that further untangling mechanisms would require a more detailed data set on land-cover history, scale and context. Ehrmann et al. [76] concluded that historical forest continuity on its own was not significant but considering the resulting functionality of the forest stand was important. Few authors [52,77] considered the temporal forest successions by describing the forest as a gradient from monoculture or non-forested areas to second growth forest and to old growth or pristine forest. Both studies took place in North America and both showed that infection prevalence or vector abundance was positively associated with an intermediate stage (second growth forest in regeneration after habitat change or pine-mixed hardwood forest). Our scoping review showed that matching heterogeneous spatial and temporal scaled data is challenging and rarely done yet promising.

### 4.3 Challenges in assessing forest cover dynamics: complexities between typology and successional stages

The forest cover dynamics keywords found, such as deforestation, fragmentation, conversion and reforestation, were consistent with the literature and succeeded in encompassing many articles in the scope. However, this typology may lead to some biases. Thinking in terms of forest stages opens new perspectives in terms of forest functionality. For example, a fragmented forest is not necessarily synonymous of a habitat loss as it can be described in a context of reforestation even if the total habitat amount increases [6]. Reforestation can also result in a second growth forest, with an intermediate perturbed area, favouring generalist species and potentially vector-borne diseases. This idea has been modelled by McClure and Diuk-Wasser [68] and empirically studied by several authors [67,78], who found a nonlinear quadratic response of forest cover dynamics on disease risk. The quadratic response suggests a threshold of forest quantity or maturity beyond which biodiversity begins to limit disease transmission. Such a response has been observed in forest pest invasion [79]. This threshold is difficult to evaluate in terms of forest stage/age. We also found in this scoping review that reforestation and conversion were underrepresented in the sampled articles. If deforestation is quite easy to detect as it is often a local and sharp change, natural reforestation dynamics can be very challenging to spot on maps and remain unnoticed because of their long and gradual process. Spontaneous forest and tree plantations for tree products such as oil palm or timber are also sometimes grouped into a single category. Conversion might be temporarily framed as deforestation if primary forest was removed and then turned into plantation. Depending on the data resolution, conversion could also be framed as reforestation or fragmentation if commercial species are recognized as non-exploited trees. This makes the study of conversion dynamics difficult. Behind these difficulties lies a problem of monitoring and definition [80].

### 4.4 The multifaceted nature of forests: implications for understanding acari-borne disease transmission

The absence of a universally accepted definition of a forest within the field of disease ecology posed a real challenge in assessing articles dealing with forest and Acari-borne diseases and vectors. According to Chazdon et al. [80], forest definition is closely related to the objectives of forest management. Historically, the conception of forests has been rooted in timber production, with conservation goals being integrated in the 1960s thanks to growing environmental concerns. Later, climate change awareness revealed the carbon storage potential of forests and their role in climate change mitigation. In 2012, the creation of the Intergovernmental Panel on Biodiversity and Ecosystem Services eventually expanded the concept of forests as providers of multiple ecosystem services. The time range of our sample aligned quite well with the emergence of this late concept as the majority of the articles were published between 2013 and 2020. This evolution has led to the coexistence of multiple international definitions, formulated by various environmental and forestry organizations such as the United Nations Food and Agriculture Organization (FAO), the United Nations Framework Convention on Climate Change (UNFCCC), the United Nations Convention on Biological Diversity (UN-CBD), the United Nations Convention to Combat Desertification (UN-CCD) and the International Union of Forest Research Organizations (IUFRO). The FAO definition was the earliest and most widely used [81]. FAO defined forest as “land spanning more than 0.5 ha with trees higher than 5 m and a canopy cover of more than 10 percent, or trees able to reach these thresholds in situ. It does not include land that is predominantly under agricultural or urban land use.” [20]. Consistent with that statement, very few authors in the sampled articles proposed a definition of forest and those who did consistently used the FAO definition.

Chazdon et al. [80] explained that tree plantation falling within the FAO definition of forest may lead to misuse when states and governments use it to meet ambitious global restoration goals. However, even tree covers with limited ecological value might be relevant regarding the risk of Acari-borne disease and should be considered. Differences in functional, structural and compositional properties might have an effect on vectors as suggested by the results and discussion of this scoping review. Forest conversion, and thus tree plantation, is an important section of the literature that must be considered when focusing on host, vector and pathogen dynamics. Recognizing this, the studies gathered in our scoping review included all types of forest worldwide in a very wide range of ecosystems with different structures and functionalities. The lack of a common description of forest or the tools available to the authors (such as maps) made it difficult to classify and summarize the overall effects of forests on the pathogens or the vectors. Forest area was the best common descriptor to summarize the results but may have prevented consensus and identification of risk factors. We need consensus on the concepts and definition of forest that encompass tree composition, structure, configuration and functionalities. Such common and detailed descriptors would enable us to understand what the risk factors may be and then understand how to manage them for the vector-borne disease prevention.

### 4.5 Perspectives

Surprisingly, studies on scrub typhus were few in our dataset. We made a complementary search to assess the lack of studies investigating forests, chigger mites and scrub typhus. We confirmed that this Acari-borne disease was much more associated with keywords like fallow, shrubland or bush. These habitats can be seen as intermediate stages or as ecotones in forest evolution and dynamics [82]. The results of Morand and Lajaunie [83] showed that it is not meaningless to study them under this scope. Our scoping review provides a new perspective for research exploring scrub typhus in a context of forest cover dynamics. No studies have specifically addressed the predator-prey relationship within the context of forest cover dynamics. Although important studies explored the balance of these interactions [23,84,85], they did not consider the impact of landscape dynamics. Coupling forest cover dynamics with community dynamics and, more broadly, integrating trophic networks remains to be done, which should be addressed in future research. Lastly, there is the lack of conversion and deforestation in a temperate context and reforestation and fragmentation in tropical context. This scoping review showed a strong geographical determination of forest cover dynamics partly due to the global trend of forest cover change [16].

### 4.6 Relevance

Exploring the state of knowledge on forest cover dynamics and Acari-borne diseases is a topical issue because, in a very changing world, forest cover is also undergoing an important process of change. Winkler et al. [82] as well as Grantham et al. [16] observed a net gain of forest in the northern part of the world and a net loss in the southern part as well as a disparate level of functional integrity. Ecological, social and political drivers lead those changes. Among these changes, new agricultural practices like agroforestry or agricultural abandonment that create unsettled new areas favour shrubland encroachment. These intermediate stages can eventually evolve back to forest [82]. On the contrary, agricultural extension producing international goods (soybean, beef, sugar cane, oil palm and cocoa) cause major deforestation [82]. Environmental policies also impact forest cover. The REDD + initiative (Reducing Emissions from Deforestation, forest Degradation, and other forest activities) leading to reforestation has been internationally supported by the Paris Agreement with the objective of carbon reduction up to −1.1 ± 0.5 GtCO_2_e/yr fixed at 2030 [86]. Climate change is also causing a significant and yet hard to predict forest cover change (range shift, drought, pest vulnerability, increase of exceptional event frequencies like storm, fires, etc) [87]. This makes the study of forest cover dynamics more relevant than ever.

### 4.7 Limitations

Our scoping review encompassed only articles written in English which led to an underrepresentation of non-English speaking countries (especially Chinese, Spanish, French, Russian, and Japanese). Very few studies addressed chigger-borne diseases in relation to reforestation or conversion, which made it hard to draw conclusions on the link between forest cover dynamics and these diseases. Finally, as already explained, the forest descriptions and definitions available along with the very varied contexts in the collected articles rendered difficult to statistically test the impact of forest cover dynamics on Acari-borne risk. More broadly, the scoping review with its methodology could not bring evidence of a particular effect or intervention as pointed out by Arksey and O'Malley [19]. It rather aimed to summarize and disseminate research findings and to identify research gaps. Although we are not specifically testing a certain impact of forest cover dynamics, this scoping review can still be seen as a first step to check full systematic review feasibility.

## 5 Conclusion

As far as we know, no previous literature review performed such a comprehensive analysis of the links between forest cover dynamics and Acari-borne diseases worldwide. This article provides a broad picture of forest cover variations and impacts on the presence of vectors and pathogens and how forest ecosystem, wildlife, vectors, pathogens and humans are intertwined. Landscape ecology coupled with a systemic and transdisciplinary approach appears promising in this field of research. Such studies could facilitate decision-making both in the field of public health and biodiversity conservation.

## CRediT authorship contribution statement

**Nolwenn Blache:** Writing – review & editing, Writing – original draft, Validation, Methodology, Formal analysis, Data curation, Conceptualization. **Karine Chalvet-Monfray:** Writing – review & editing, Validation, Supervision, Methodology, Data curation, Conceptualization. **Christophe Déprés:** Writing – review & editing, Validation, Supervision, Methodology, Conceptualization. **Serge Morand:** Writing – review & editing, Validation, Supervision, Methodology, Conceptualization.

## 6 Supplementary information

Additional file 1: Text S1. Preliminary literature review. Text S2. Data extraction form.

Additional file 2: Dataset S2. Clean raw data obtained from the scoping review methodology on the impacts of forest cover dynamics on Acari-borne disease. Dataset S3 Variable dictionary for extracted data.

Additional file 3: Fig. S4. Venn diagrams of the distribution of pathogen, human, wildlife and vector in (A) all the selected articles (n = 111) and (B) in the empirical or modelling articles only (n = 86). Fig. S5. Upset plot of the studied wildlife distribution for literature synthesis articles only (n = 24). Fig. S6. Distribution of forest impacts on the vector presence when considering empirical or modelling articles n = 86(A) and when considering literature synthesis n = 25 (B)

Additional file 4: Dataset S7. Dictionary of forest variables described in Table 5.

## Ethics statement

Review or approval by an ethics committee was not needed for this study because no data on patients or experimental animals was produced in the review article. Informed consent was not required for this study because no clinical data was produced in the review article.

## Data availability statement

All data generated or analysed during this study are included in this published article and its supplementary information files.

## Funding

This research has been funded by 10.13039/501100011073VetAgro Sup (doctoral grant, decision by the general management following the scientific council meeting dated June 14, 2022) and Clermont Auvergne Metropole, France (decision dated June 24, 2022). As a funder, Clermont Auvergne Metropole has played no role in the research.

## Declaration of competing interest

The authors declare that they have no known competing financial interests or personal relationships that could have appeared to influence the work reported in this paper.
