# Supplementary material for: A scoping review of the impacts of forest cover dynamics on acari-borne diseases: Beyond forest fragmentation
Source: Heliyon. 2025 Jan 11;11(2):e41893. doi: 10.1016/j.heliyon.2025.e41893 (PMC11787481; doi:10.1016/j.heliyon.2025.e41893)
Supplement: Multimedia component 1 [file mmc1.docx]

Additional file 1

**Text S1. Preliminary literature review**

We started our scoping review by a preliminary literature examination to refine the research question and to properly design the search strategy. We combined broad key words like “forest”, “infectious disease*”, “vector-borne disease*”, “ecosystem service*”, “biodiversity”, “dilution effect” and “ecohealth” to consider the links between forest and infectious disease. This work corresponded to the first stage: Identifying the research question formulated by Arksey and O’Malley [18]. At this stage, we were aware that “forest” could encompass various approaches regarding infectious diseases. We also recognised the need to determine what kind of infectious diseases to examine. Forest dynamics appeared to be important facets of the question. From this work, we made a list of forest dynamics encountered in the scientific articles: deforestation, fragmentation, conversion and reforestation. Once we had a general sense of the volume and scope of this field, we decided to narrow the focus on acari-borne diseases that are relevant to the question and that represent a reasonable number of articles.

**Text S2. Data extraction form.**

General information:

- Author(s):
- Year of publication:
- Study location:
- Journal:

Studied objects:

- What disease(s) is/are studied? (Zoonotic/vector-borne)
- What is(are) the vector(s)?
- What is(are) the pathogen(s)?
- What type of forest is studied? (Tropical/temperate)
- What definitions of forest did they use?
- Did they examine forest dynamics such as reforestation/deforestation/conversion/fragmentation? (noted by the authors)
- Did they consider wildlife along with the forest? Which kind?
- Did they consider humans along with the forest? Which status?

Study purpose:

- What question does the article address?
- Did the author examine the effect of the forest?
- What did the author not examine?
- Did they examine the effect of the forest on pathogen presence/prevalence?
- Did they examine the effect of the forest on vector abundance/density?

Methodology:

- What is the study's nature?
- What is the methodology?
- What are the variables (if any) used to describe the forest?
- What scales (temporal and spatial) are used?

Results:

- What are the important results?
- What effect did the evolution of forest surface have?
- What are the limitations of the study as noted by the authors?
