# Supplementary material for: A scoping review of the impacts of forest cover dynamics on acari-borne diseases: Beyond forest fragmentation
Source: Heliyon. 2025 Jan 11;11(2):e41893. doi: 10.1016/j.heliyon.2025.e41893 (PMC11787481; doi:10.1016/j.heliyon.2025.e41893)

Additional file 3:

**Figure S4.** **Venn diagrams of the distribution of pathogen, human, wildlife and vector in (A) all the selected articles (n=111) and (B) in the empirical or modeling articles only (n=86).**


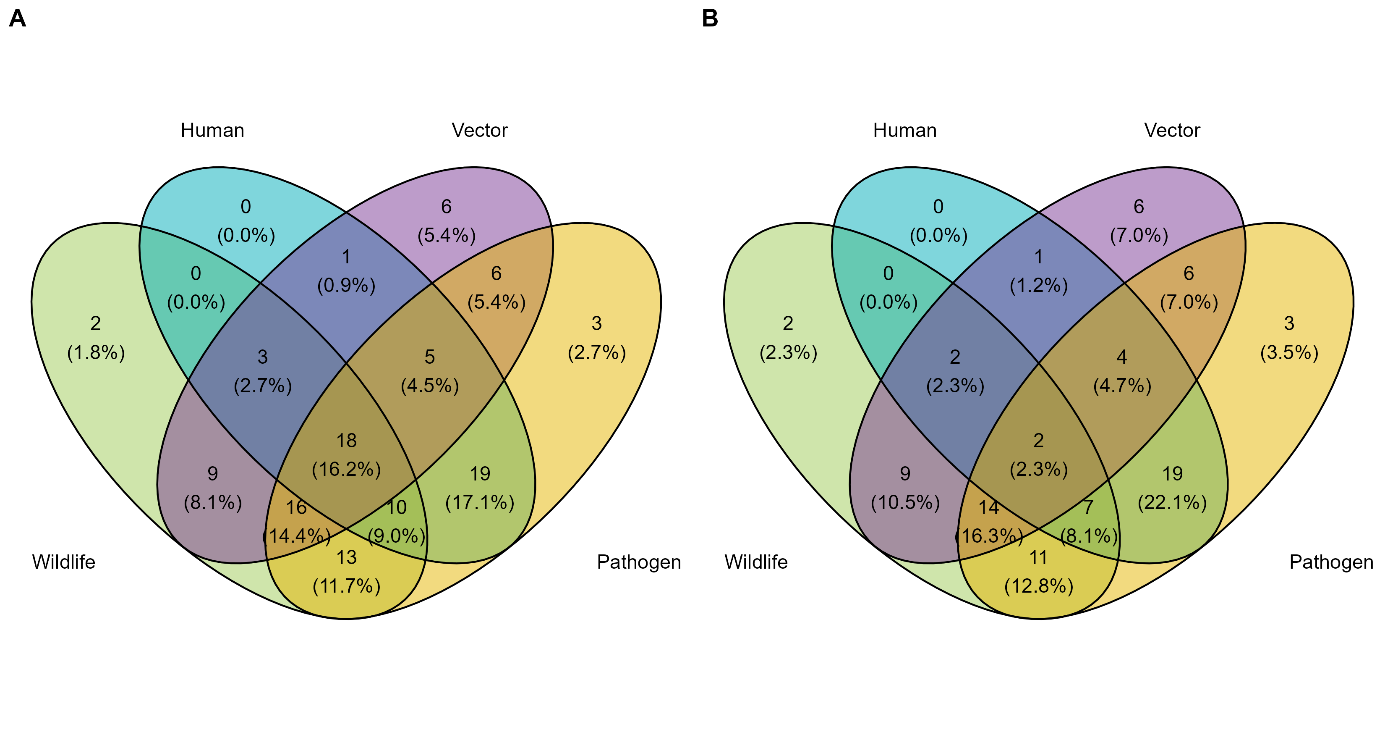


**Figure S5.** **Upset plot of the studied wildlife distribution for literature synthesis articles only (n=24).**

**
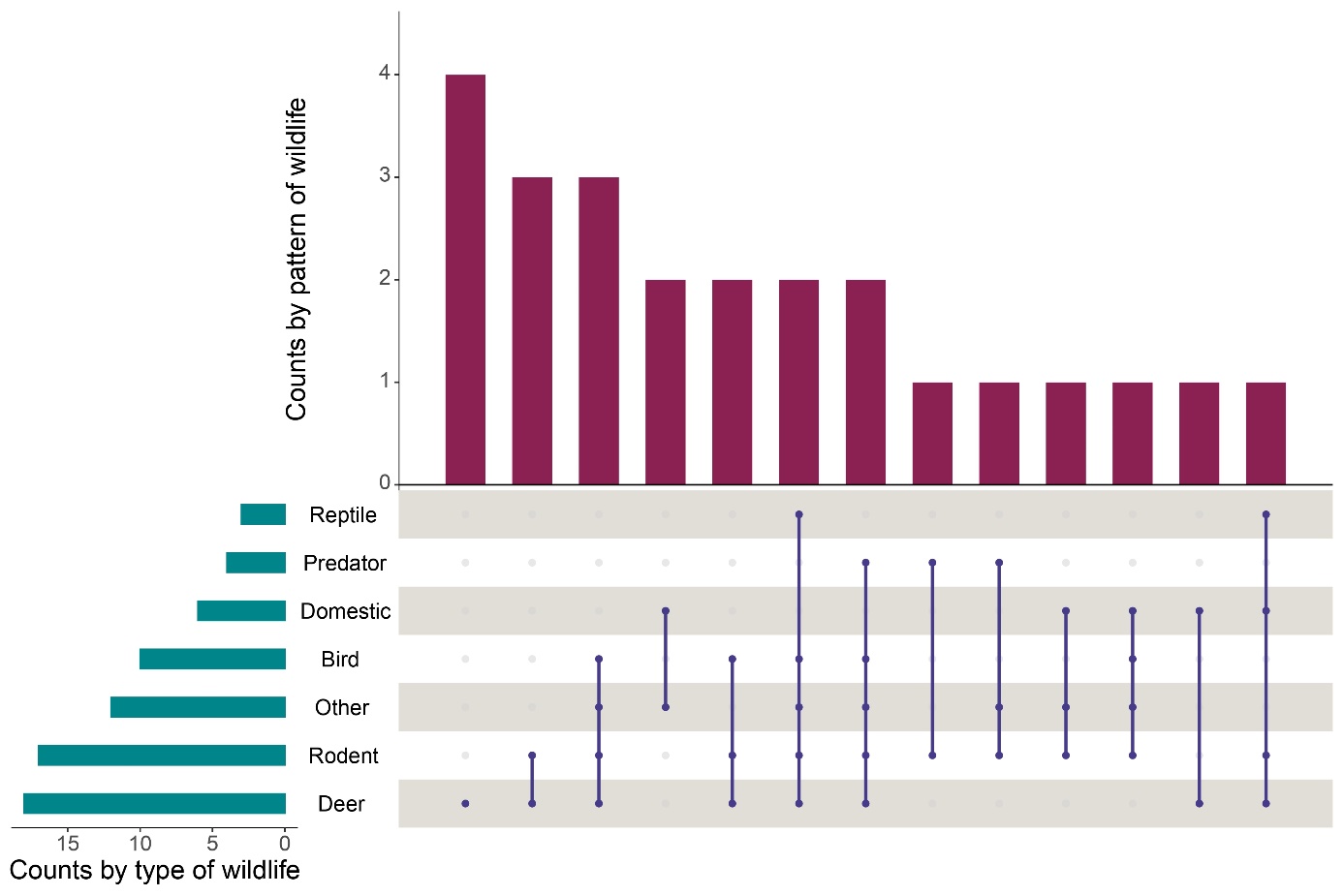
**

**Figure S6** **Distribution of forest impacts on the vector presence when considering empirical or modeling articles n=86(A) and when considering literature synthesis n=25 (B)**


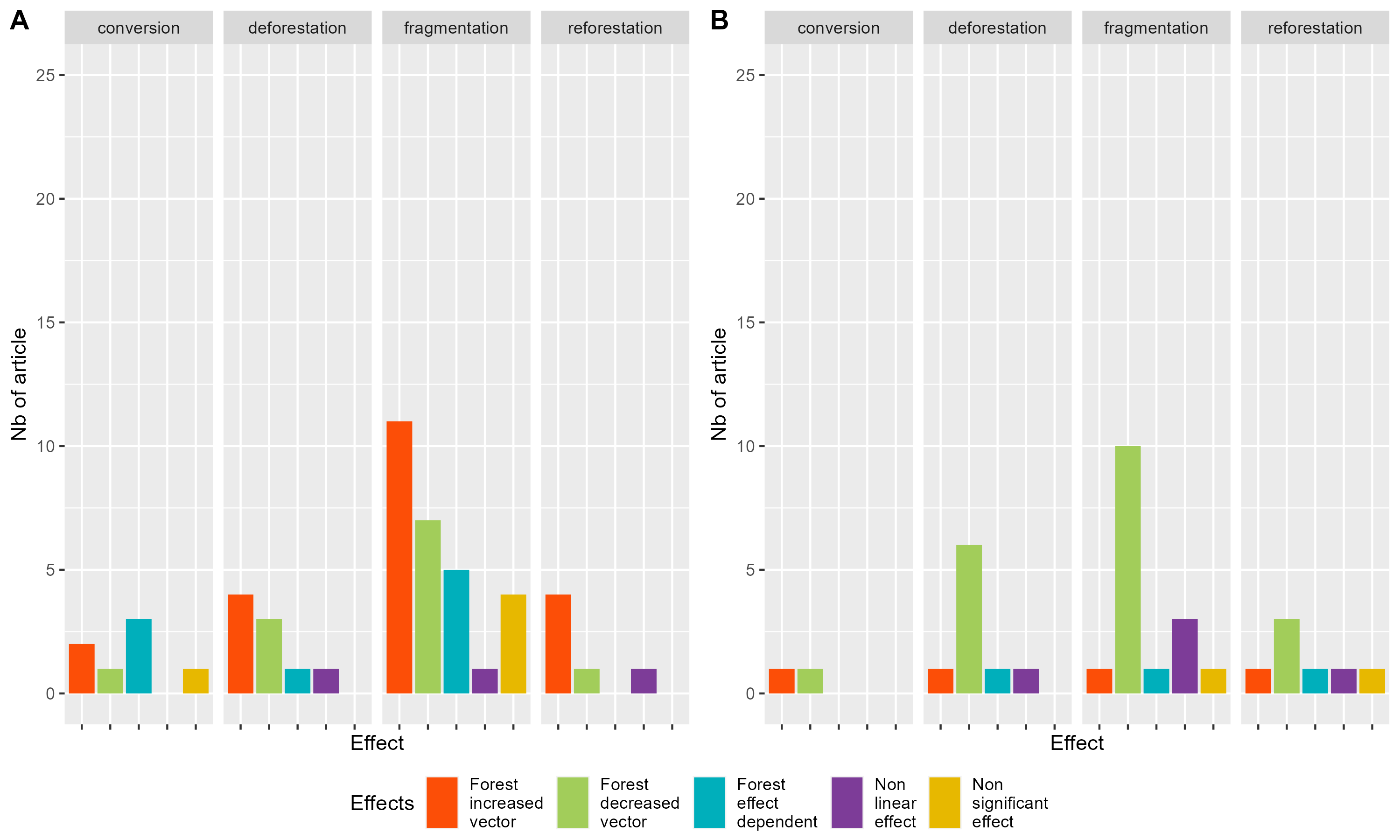

Supplement: Multimedia component 3 [file mmc3.docx]
